# CAF01 Potentiates Immune Responses and Efficacy of an Inactivated Influenza Vaccine in Ferrets

**DOI:** 10.1371/journal.pone.0022891

**Published:** 2011-08-05

**Authors:** Cyril Jean-Marie Martel, Else Marie Agger, Julie Juul Poulsen, Trine Hammer Jensen, Lars Andresen, Dennis Christensen, Lars Peter Nielsen, Merete Blixenkrone-Møller, Peter Andersen, Bent Aasted

**Affiliations:** 1 Department of Veterinary Disease Biology, Faculty of Life Sciences, University of Copenhagen, Copenhagen, Denmark; 2 Department of Infectious Disease Immunology, Statens Serum Institut, Copenhagen, Denmark; 3 National Influenza Laboratory, Statens Serum Institut, Copenhagen, Denmark; Institut Pasteur, France

## Abstract

Trivalent inactivated vaccines (TIV) against influenza are given to 350 million people every year. Most of these are non-adjuvanted vaccines whose immunogenicity and protective efficacy are considered suboptimal. Commercially available non-adjuvanted TIV are known to elicit mainly a humoral immune response, whereas the induction of cell-mediated immune responses is negligible. Recently, a cationic liposomal adjuvant (dimethyldioctadecylammonium/trehalose 6,6′-dibehenate, CAF01) was developed. CAF01 has proven to enhance both humoral and cell-mediated immune responses to a number of different experimental vaccine candidates. In this study, we compared the immune responses in ferrets to a commercially available TIV with the responses to the same vaccine mixed with the CAF01 adjuvant. Two recently circulating H1N1 viruses were used as challenge to test the vaccine efficacy. CAF01 improved the immunogenicity of the vaccine, with increased influenza-specific IgA and IgG levels. Additionally, CAF01 promoted cellular-mediated immunity as indicated by interferon-gamma expressing lymphocytes, measured by flow cytometry. CAF01 also enhanced the protection conferred by the vaccine by reducing the viral load measured in nasal washes by RT-PCR. Finally, CAF01 allowed for dose-reduction and led to higher levels of protection compared to TIV adjuvanted with a squalene emulsion. The data obtained in this human-relevant challenge model supports the potential of CAF01 in future influenza vaccines.

## Introduction

Efforts to prevent or minimize the impact of seasonal influenza in the second part of the 20th century have focused on the use of vaccines [1]. Due to the yearly changes in viral antigenic configuration [2] and the lack of carry-over protection from year to year [3], vaccination campaigns annually require a huge logistic effort to ensure that the production and delivery of the seasonal vaccine is sufficient for high population coverage [4]. In addition, the time span between the selection of the vaccine strains to the vaccine being commercially available is between 6 and 8 months [5]. In the case of pandemic avian influenza, the world population would be considered immunologically naïve, which would imply that a large part of the population should be vaccinated twice [6]. Given the current production capacities and limitations, such a demand could not be fulfilled on time, and even a single vaccination world-wide would not be realistic [7]. Additionally, the protection provided by current non-adjuvanted influenza vaccines is short-lived and declines after six months. This means that such a vaccine most likely would not be able to protect individuals against the second wave of flu sometimes observed in pandemics [8]. Also, the quality of the immune response conferred by the available killed virus vaccines has been debated and is almost solely focused on a humoral response directed against highly variable surface proteins [9], whereas the induction of cell-mediated immune (CMI) responses is negligible [10], [11]. In contrast, it has been shown during natural infections that T-cells target primarily conserved proteins from the inner part of the virion that may mediate cross-protection against heterologous strains [12] and long-lived protection [13]. Protection relying solely on antibodies, as induced by the most common inactivated vaccines, is short-lived and falls below effective levels after 6 to 12 months, especially in the elderly. Therefore, the ideal vaccine to fight both epidemic and pandemic influenza should induce both a humoral and a cellular immune response with only one injection of a minimal dose [14].

Several strategies have been considered to remediate the shortcomings of non-adjuvanted influenza vaccines. Live-attenuated vaccines (LAIV) for intra-nasal immunization have been commonly used in some parts of Europe, and FluMist became in 2003 the first LAIV available outside of Europe. However, LAIV are not recommended for children under 2 or adults over 50, which are the two populations the most at risk for severe influenza. Other mucosal routes of immunization, such as oral [15] or sublingual [16], have also been considered, but the few vaccines that have reached the market are still treated with caution [17]. Mucosal tolerance remains a hurdle when it comes to designing new mucosal vaccines not relying on live vectors. The addition of an adjuvant to the TIV to improve its immunogenicity is another favoured strategy. A recent study showed high antibody titers after injection of an aluminium-adjuvanted vaccine [18]; however this type of adjuvant (reviewed in [19]) has shown little or no benefits in most other studies [20], [21]. Although it is the only adjuvant available for world-wide usage in humans, it is generally accepted that novel and more effective adjuvants should be employed for influenza vaccines. Water-in-oil emulsion adjuvants have been widely used in experimental and commercial influenza vaccines, with MF59 being the first emulsion licensed for human use in some European countries. A number of other oil-in-water emulsions are currently in the pipeline for both pandemic and pre-pandemic influenza vaccines, and GlaxoSmithKline recently developed a pandemic and a pre-pandemic avian influenza vaccine, both using the oil-in-water emulsion AS03, and both being approved in Europe since May 2008 [22]. However, this adjuvant may not be the most efficient way of inducing strong Th1 responses and alternative vaccination strategies, e.g. the use of other CMI-inducing adjuvants or combination adjuvants supplementing emulsions with immunomodulators like CpG or MPL [23], remain therefore a highly prioritized research area.

In this study, the effect of a novel combination adjuvant, cationic adjuvant formulation 01 (CAF01), in a conventional TIV against influenza was investigated. This new adjuvant based on cationic dimethyldioctadecylammonium (DDA) and trehalose 6,6′-dibehenate (TDB) induces both a strong T-cell response and a concomitant antibody response. Similar promising results for CAF01 have been described in other models, including malaria, chlamydia and tuberculosis [24], [25]. The studies presented here were performed using ferrets for testing both immunogenicity and protection.

Although considered the preferred animal model for use within the field of influenza, the lack of ferret-specific reagents has limited the use of relevant *in vitro* assays. Herein, we used the recently developed panel of reagents including antibodies against, Immunoglobulin subclasses, cytokines and T cell subsets [26] for monitoring immune response induced by a CAF01-adjuvanted influenza vaccine. The adjuvant was found to increase the levels of IgG and IgA antibodies to influenza proteins in nasal washes and serum, and the functionality of those antibodies was confirmed by a hemagglutination inhibition assay. In addition, intracellular staining of interferon-gamma (IFN-γ) demonstrated the induction of T-cell responses upon vaccination and subsequent influenza challenge. Along with the highly elevated immune responses, CAF01 led to a significantly accelerated reduction in the viral load compared to the un-adjuvanted split vaccine, even when using a low dose of 0.15 µg split vaccine. CAF01 also produced higher IgG titers and led to a lower shedding of viral particles in infected animals compared to an emulsified squalene adjuvant.

## Materials and Methods

### Ethics statement

All experiments were approved by the Danish Animal Care and Ethics Committee and conducted in accordance with the Danish Animal Experimentation Act and the European Convention for the Protection of Vertebrate Animals used for Experimental and Other Scientific Purposes (permit nr 2006/561–1105).

### Vaccine and adjuvant

The CAF01 adjuvant was used in a dosage of 1250 µg DDA and 250 µg TDB from Avanti Polar Lipids (Alabaster, AL) prepared as previously described [25]. The squalene emulsion was used in a dosage of 4.85 mg DL-α-tocopherol , 11.86 mg Polysorbat 80 and 10.68 mg squalene from Sigma-Aldrich (Steinheim, Germany) as previously described for the adjuvant in the Prepandrix vaccine [27]. The formulation was emulsified using a IKA T25 digital ULTRA-TURRAX® high shear mixer (Staufen, Germany) under 6,000 rpm for 5 minutes in order to provide a homogeneous feedstock for final emulsification [28]. The final squalene emulsion was generated by ten serial passages of emulsion through a polycarbonate membrane from Avestin (Mannheim, Germany) with a pore diameter of 200 nm. Sanofi-Pasteur's Vaxigrip TIV was purchased from a commercial distributor. The two vaccines used in this study were the 2005/2006 formulation containing 15 µg of hemagglutinin (HA) from A/New Caledonia/20/99 (H1N1), 15 µg of HA from A/New York/55/2004 (H3N2) and 15 µg of HA from B/Jiangsu/10/2003; and the 2008/2009 formulation containing 15 µg of HA from A/Brisbane/59/2007 (H1N1), 15 µg of HA from A/Brisbane/10/2007 (H3N2) and 15 µg of HA from B/Florida/4/2006.

### Immunization schedule

All experiments were performed on outbred, 6 to 10 months old, female ferrets (*Mustela putorius furo*) obtained from Møldrup fur farm (Møldrup, Denmark). The ferrets were housed in a class II isolation facility at the Faculty of Life Sciences, University of Copenhagen, with free access to food and water. Prior to vaccinations, animals were confirmed to be seronegative for circulating influenza A (H1N1 and H3N2) and influenza B viruses by haemagglutination inhibition assay (HAI) and ELISA. For the A/New Caledonia/20/99 (H1N1) challenge study, ferrets (n = 8) were immunized intra-muscularly (i.m.) in the hind leg with CAF01 (250 µl) adjuvanted or non-adjuvanted Vaxigrip (80 µl, containing 2.5 µg of each HA) twice at week 0 and 2 weeks after. A control group of unvaccinated animals received 250 µl of PBS per injection instead of the vaccine. 6 weeks after vaccination, all animals were inoculated intranasally (i.n.) with 10^7^ TCID_50_ of A/New Caledonia/20/99 (H1N1) produced in eggs. This study was performed three times with the same design. For the A/Brisbane/59/2007 (H1N1) dose-response experiment, ferrets (n = 4) were immunized twice i.m. at week 0 and week 2 with one of three doses (0.5 ml of vaccine/15 µg of each HA, 50 µl of vaccine/1.5 µg of each HA, or 5 µl of vaccine/0.15 µg of H1) with or without the CAF01 adjuvant (250 µl). A control group received only PBS. At week 6, all animals were inoculated i.n. with 10^7^ TCID_50_ of A/Brisbane/59/2007 (H1N1) produced in eggs. During challenge, in both studies, nasal washes were taken daily from day 0 to day 6 post infection (p.i.). Blood samples were taken from the cranial vena cava at day 3, 5, 7 and 10 p.i. For the squalene emulsion vs CAF01 head-to-head comparison, ferrets were immunized twice i.m in the hind leg at week 0 and week 4 with 0.5 ml of TIV with CAF01, a squalene emulsion, or without adjuvant. A control group of mock-vaccinated animals received only PBS. At week 6, all animals were inoculated i.n. with 10^7^ TCID_50_ of A/Brisbane/59/2007 (H1N1) produced in eggs.

### Viral excretion in nasal washes

Nasal washes were performed using a pipette by application of 1 ml of PBS into the nostrils of each ferret. Subsequently, the animals sneezed and the expelled material (nasal wash sample) was collected and kept at −80°C.

The concentration of viral RNA was measured by real time RT-PCR using primers and probe from the matrix gene (available on request). RNA was extracted from the samples using the total nucleic acid kit on the semi automatic Magnapure extraction machine from Roche (Hvidovre, Denmark). The eluted RNA was analyzed in one-step RT-PCR using the RT-PCR One-step kit from Qiagen (Copenhagen, Denmark). The real-time PCR assays were performed on an MX3005 thermocycler from Stratagene (LaJolla, CA). For every assay, a standard curve with titrated A/New Caledonia/99 (H1N1) or A/Brisbane/59/2007 (H1N1) influenza virus was used to calculate the relative amount of viral RNA present in the sample.

### Influenza-specific IgG and IgA ELISA

For IgG titration, Maxisorp plates (NUNC, Roskilde, Denmark) were coated at 4°C overnight with Vaxigrip (1 µg hemagglutinin from H1N1 per ml) in carbonate buffer pH 9.6. The plates were washed three times with PBS containing 0.05% Tween-20 and blocked with PBS with 1% BSA for two hours. The plates were washed and 100 µl of serial dilutions of serum sample were tested in duplicates. After a one hour incubation period followed by washes, 150 µl of biotinylated polyclonal rabbit anti-mink IgG antibody [26] diluted 1∶500 was added and the plates were incubated for one hour. After thorough washes, 100 µl of HRP-streptavidin (Dako, Denmark) was added followed by incubation for 30 minutes at room temperature and subsequent development of the reaction with OPD tablets (1,2-phenylendiamin-dihydrochlorid, Dako, Denmark) following the manufacturer's instructions. IgA levels in nasal washes were investigated in a similar fashion as for the above mentioned IgG ELISA, except that a HRP-conjugated anti dog IgA (AbD-Serotec, Denmark) polyclonal antibody was used instead of the anti-IgG [26]. Antibody titers are expressed as the highest dilution with an optical density (OD) reading greater than 2 times the mean OD + standard deviation of similarly diluted negative control samples.

### FACS analysis of peripheral blood leucocytes

Staining was performed as previously described [29] with the following modifications. Approximately 2 million peripheral blood leucocytes (PBLs) prepared after hypotonic lysis of erythrocytes with 0.15 M NH_4_Cl were cultured in 1 ml of modified RPMI containing 20 mM Hepes and L-Glutamine (Sigma, St. Louis, USA) 10% FCS (fetal calf serum), 100 IU/mL penicillin and 100 µg/mL streptomycin. For non-specific stimulation of lymphocytes, the cultures were incubated for 4 hours with a medium containing brefeldin A (Sigma, St. Louis, USA) to a final concentration of 10 µg/ml culture, ionomycin (Sigma, St. Louis, USA) to a final concentration of 1 µg/ml and phorbol-12-myristate-13-acetate (PMA, Sigma, St. Louis, USA) to a final concentration of 20 ng/ml. For the antigen-specific stimulation, the PBLs were cultured 24 hours with medium containing 1 µg/ml of recombinant H1 hemagglutinin from A/New Caledonia/20/99 (Protein Sciences Corporation, CT, USA). After culture, the PBLs were fixed in 4% paraformaldehyde and permeabilized with 0.1% saponin (Sigma, St. Louis, USA) and stained with 15 µl of PE-conjugated ferret cross-reactive mouse monoclonal antibody to bovine IFN-γ (clone CC302, AbD-Serotec, Denmark) [26]. Finally, the cells were analyzed with a Becton Dickinson Calibur flow cytometer. Gating for lymphocyte populations was done as previously described [30].

### Hemagglutination inhibition (HAI) assay

Hemagglutination inhibition assay was performed according to the standard WHO protocol WHO/CDS/CSR/NCS 2002.5 Rev.1 [31]. Hemagglutination was measured by the viral agglutination of 0.4% (vol/vol) guinea pig red blood cells (Statens Serum Institut, Denmark). Serum samples were incubated overnight at 37°C with 4 parts receptor destroying enzyme (RDE) to destroy nonspecific inhibitors of hemagglutination. The reaction was stopped by denaturing the enzyme at 56°C for 30 minutes. RDE-treated sera were two-fold serially diluted in 96-well v-bottomed microtiter plates (Nunc, Roskilde, Denmark), and an equal volume of virus adjusted to 8 hemagglutination units was added. HAI titers were determined by the reciprocal dilution of the last well which contained non-agglutinated red blood cells.

### Statistical analysis data

HAI and ELISA data are given as geometric mean titer (GMT) with 95% confidence interval. FACS and RT-PCR data are given as arithmetic mean ± SEM. Statistical significance was asserted by a two-way analysis of variance (ANOVA) followed by Bonferroni's *t*-test, except for the dose-reduction experiment, where a one-way analysis of variance (ANOVA) was applied, followed by Dunnett's *t*-test. All tests were performed on log-transformed data, using GraphPad Prism version 5.0 (La Jolla, CA). The following conventions were used: *p<0.05, ** *p*<0.01, *** *p*<0.001.

## Results

### CAF01 enhances both antibody and CMI responses of the TIV

**Figure 1:**
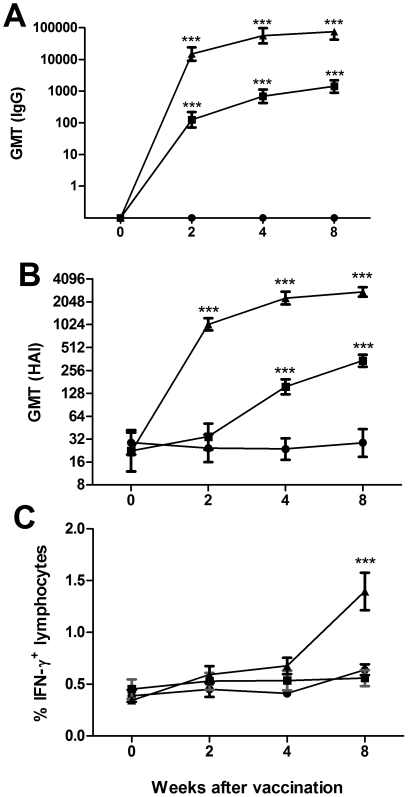
Vaccine-specific immune responses. 6 to 10 months-old ferrets were immunized twice at two week-intervals with CAF01 adjuvanted (▴) and non-adjuvanted (▪) influenza vaccine Sanofi-Pasteur's Vaxigrip season 2005/2006 (n = 8 per group). A third group was mock-vaccinated (•). **A.** Serum IgG antibodies against the vaccine antigens measured by IgG specific ELISA at various timepoints. The titer was defined as the reciprocal value of the highest positive dilution. **B.** Hemagglutination inhibition assay using influenza A (New Caledonia 1999 (H1N1). HI titers were determined by the reciprocal dilution of the last well which contained non-agglutinated red blood cells. All ELISA values are expressed as geometric mean titers (GMT). **C**. Percentage of IFN-γ-positive lymphocytes: peripheral blood leucocytes were isolated and stimulated overnight with a recombinant H1 hemagglutinin from A/New Caledonia/20/99 (H1N1). Values marked with an asterisk are significantly different (*, *p*<0.05; **, *p*<0.01; ***, *p*<0.001), assessed by ANOVA.

Prior to vaccination, all ferrets were tested negative for influenza vaccine-specific IgG antibodies in serum by ELISA, and their baseline virus hemagglutination inhibition capacity was assessed. Ferrets were vaccinated twice with a dose of TIV corresponding to 2.5 µg of hemagglutinin (H1) administered without adjuvant or with CAF01. A third group was left unvaccinated. Two weeks after the first immunization, both the groups that received the non-adjuvanted vaccine and the vaccine with CAF01 showed a significant increase in their vaccine-specific IgG antibody titers in serum (fig. 1A). However, immunization with CAF01 led to higher levels of antibody titers compared to the levels induced by the vaccine alone (*p*<0.001). These increased antibody titers persisted until week 8 with IgG antibody levels in the CAF01 group being 40-fold higher than the levels in the group receiving the vaccine only. To further analyze the functionality of the antibody responses, hemagglutinin inhibition assay was performed in parallel. As shown in figure 1B, the CAF01 group also showed a rise in HAI titers two weeks after the first immunization, whereas no significant increase was induced by the vaccine alone at that time point. Significant HI titers were not detected in the non-adjuvanted group until week 8 and were about 10 times lower than in the CAF01 group.

In order to analyse CMI responses, peripheral blood leukocytes were isolated from the blood of immunized ferrets, cultivated for 24 hours with a recombinant H1 hemagglutinin from H1N1 A/New Caledonia/20/99 and stained for intra-cellular IFN-γ. The percentage of IFN-γ-positive lymphocytes of the total number of lymphocytes was measured by flow cytometry at different time points after vaccination. The baseline of about 0.5% of IFN-γ positive lymphocytes observed in naive animals remained the same for all three groups (mock-vaccinated, vaccine only, vaccine adjuvanted with CAF01) until week 4 after vaccination. At week 8, the group that received CAF01 showed a significant increase in the percentage of IFN-γ positive lymphocytes reaching levels of approximately 1.5% positive cells (fig. 1C).

### CAF01-adjuvanted TIV leads to accelerated virus clearance

**Figure 2:**
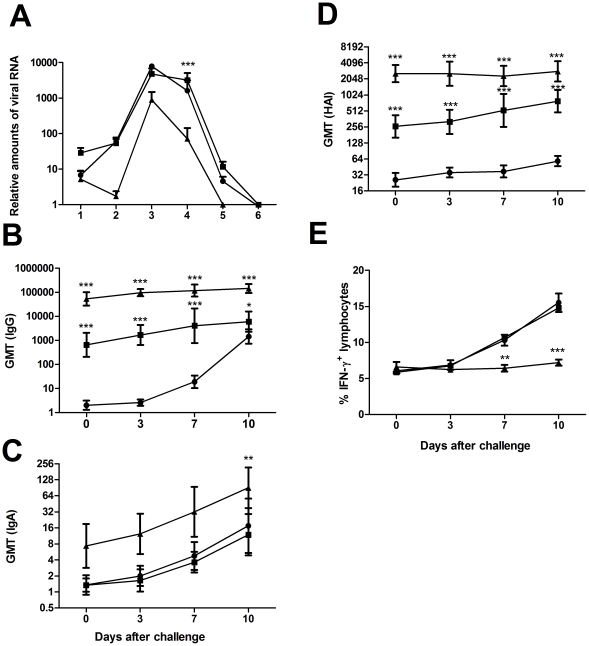
Challenge with A/New Caledonia/20/99 (H1N1). 6 to 10 months-old ferrets were immunized twice at two week- intervals with CAF01 adjuvanted (▴) and non-adjuvanted (▪) influenza vaccine Sanofi-Pasteur's Vaxigrip season 2005/2006 (n = 8 per group). A third group was mock-vaccinated (•). All ferrets were challenged with 10^7^ TCID_50_ of A/New Caledonia/20/99 (H1N1) four weeks after the second immunization (n = 8 per group). **A.** Relative amounts of viral RNA found in nasal washes of infected animals during the first six days of challenge, measured by quantitative RT-PCR. **B.** Vaccine-specific IgG titers measured in serum by ELISA **C.** Vaccine-specific IgA titers measured in nasal washes by ELISA. **D.** Hemagglutination inhibition assay titers using influenza A New Caledonia 1999 (H1N1). All ELISA values are expressed as geometric mean titers (GMT). **E.** Percentage of IFN-γ-positive lymphocytes after 4 hours stimulation with PMA and ionomycin. Values marked with an asterisk are significantly different (*, *p*<0.05; **, *p*<0.01; ***, *p*<0.001), assessed by ANOVA, except for B assessed by log-rank test.

Four weeks after the second immunization, ferrets received a H1N1 challenge through the nasal cavities. Nasal washes were performed on infected ferrets during the first five days of the challenge, and the relative amounts of viral RNA in each wash were measured by quantitative RT-PCR. As shown in fig. 2A, the group that received the vaccine adjuvanted with CAF01 showed RNA concentrations 10 to 100-fold lower than the group that received the vaccine only and the mock-vaccinated group. By measuring the percentage of animals excreting virus during infection (cumulative results of three experiments) the CAF01-vaccinated animals were found to have 10–50 times less viral load throughout the experiment and to clear the infection more rapidly than the non-adjuvanted vaccine group and the control group (fig. 2A). In contrast to the CAF01 group the mock-vaccinated animals and animals receiving vaccine alone were found to still excrete the virus at day 5.

To examine the protective immune responses during the influenza infection, ferret sera and nasal fluids were examined for antibody responses at different time points during the infection. As previously, the quantities of antibodies against influenza proteins in serum (IgG) and nasal washes (IgA) were measured by ELISA whereas the HAI was performed on serum only. As shown in figure 2B, the high titers of IgG antibodies induced by the vaccine with the adjuvant after the second immunization persisted during the challenge. Titers were significantly higher as the animals entered the infection (day 0) and remained higher at day 10 (P<0.001) in the group that received the adjuvanted vaccine compared to the group without CAF01. Low but significant titers of IgA antibodies in the nasal wash were detected at the day of inoculation in the ferrets vaccinated with CAF01 and those titers rose exponentially upon challenge (fig. 2C). In contrast, no detectable levels of IgA were found at day 0 in the group that received the vaccine only. In these ferrets, increasing but still very low levels of IgA were found throughout the 10 days of challenge with a level comparable to that of mock-vaccinated animals. Finally, HAI titers were also higher in ferrets receiving the vaccine adjuvanted with CAF01. The HAI titers were approximately 10 fold higher compared to the non-adjuvanted vaccine at day 0 after challenge inoculation, and 3 times higher at day 10 (fig. 2D). Interestingly, mock-vaccinated animals failed to show any elevation in their HAI titers during the 10 days of the challenge.

Along with the vaccine-induced immune response, we also measured the overall level of infection driven T cell activation [32], by stimulating the T cells with a polyclonal activator known to expand preferentially activated T cells (PMA and ionomycin) in a TCR-independent manner [33], [34]. The cells were subsequently stained for intra-cellular IFN-γ (fig. 2E). We used this read-out as a marker of infection. All three groups had the same baseline levels at day 0 and 3 p.i. (around 6% of IFN-γ-positive lymphocytes) but subsequently increased their level of intra-cellular IFN-γ at day 7 after challenge in mock-vaccinated animals and animals that received the vaccine alone. These two groups showed significantly higher levels of IFN-γ producing cells than the group that received the vaccine adjuvanted with CAF01 indicating a better control with viral replication and less T cell activation.

### The use of CAF01 in the influenza vaccine has a clear dose-sparing effect

**Figure 3:**
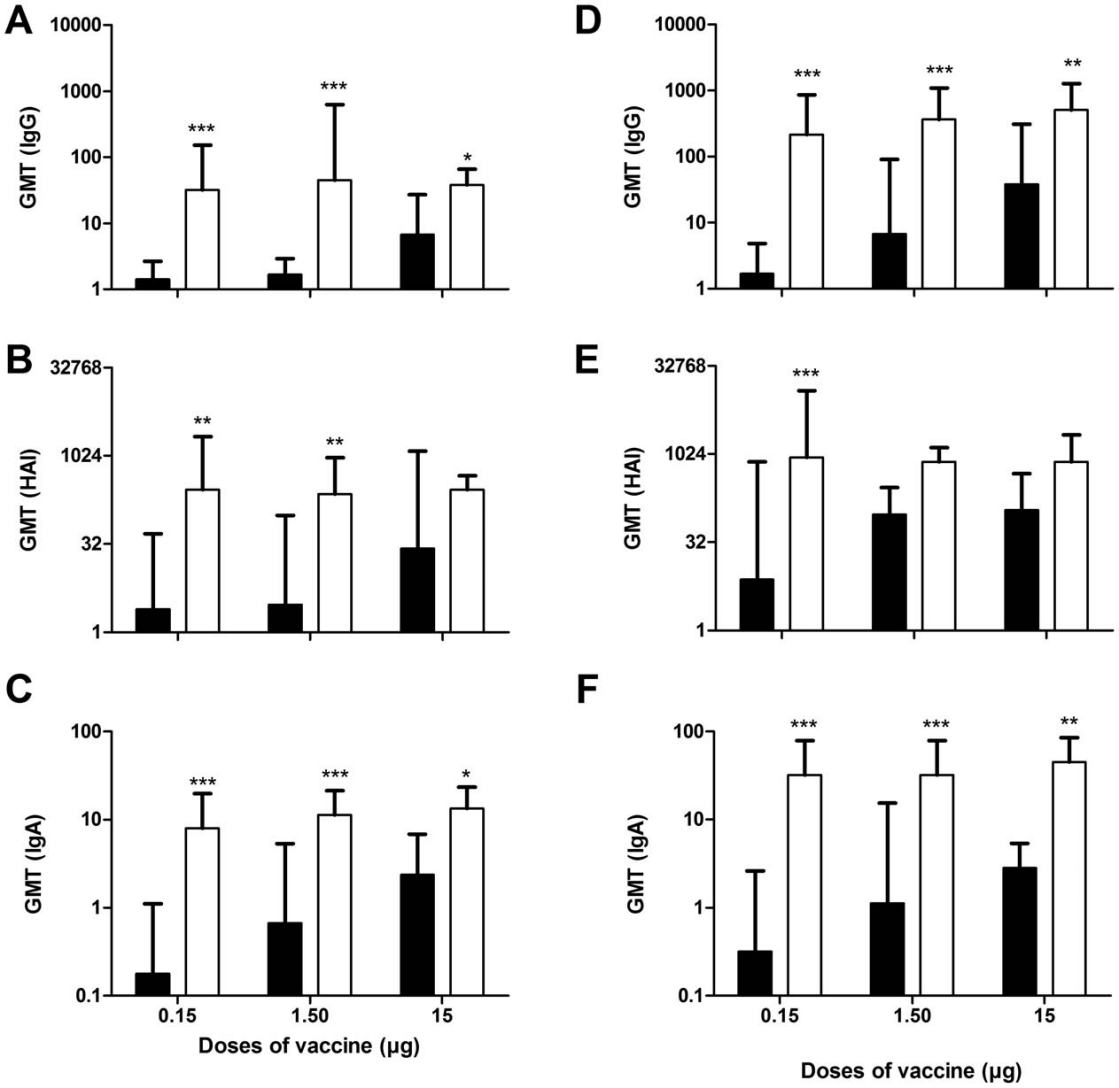
Immune responses after immunizations with different doses. 6 to 10 months-old ferrets were immunized twice at two week-intervals with different doses of CAF01 adjuvanted and non-adjuvanted influenza vaccine Vaxigrip season 2008/2009 (n = 4 per group). White bars: CAF01-adjuvanted vaccine, black bar: non-adjuvanted vaccine. A, B and C show results after 1 immunization. D, E and F show results after 2 immunizations. A and D show vaccine-specific IgG titers measured in serum by ELISA. B and E show hemagglutination inhibition assay serum titers using influenza A/Brisbane/59/2007 (H1N1). C and F show vaccine-specific IgA titers measured in nasal washes by ELISA. All ELISA values are expressed as geometric mean titers (GMT) and values marked with an asterisk are significantly different (*, *p*<0.05; **, *p*<0.01; ***, *p*<0.001), assessed by ANOVA.

In order to evaluate the dose-sparing effect of CAF01, groups of ferrets (n = 4) received three different doses of a TIV (season 2008–2009) corresponding to 15 µg, 1.5 µg or 0.15 µg, respectively, of hemagglutinin from A/Brisbane/59/2007 (H1N1). The humoral response was subsequently measured two weeks after a single vaccination (fig. 3 A,B and C) or four weeks after a booster vaccination (fig. 3D, E and F). After a single immunization, all three groups that received the vaccine with CAF01 displayed noticeable levels of antibodies measured as both IgG and HAI titers. In these groups, antibody levels were higher than in the groups that received the vaccine alone. This trend was accentuated after a booster vaccination with the three groups that received CAF01 showing significantly higher levels than the groups that received the vaccine alone. After both the single immunization and the booster did even the lowest dose of vaccine (0.15 µg) adjuvanted with CAF01 give rise to higher IgG titers than the highest dose (15 µg) of the non-adjuvanted vaccine. Although the levels of IgA in nasal washes were generally lower, the group receiving CAF01-adjuvanted vaccine again displayed levels significantly higher than their counterparts that received the vaccine alone (fig. 3C and F).

**Figure 4:**
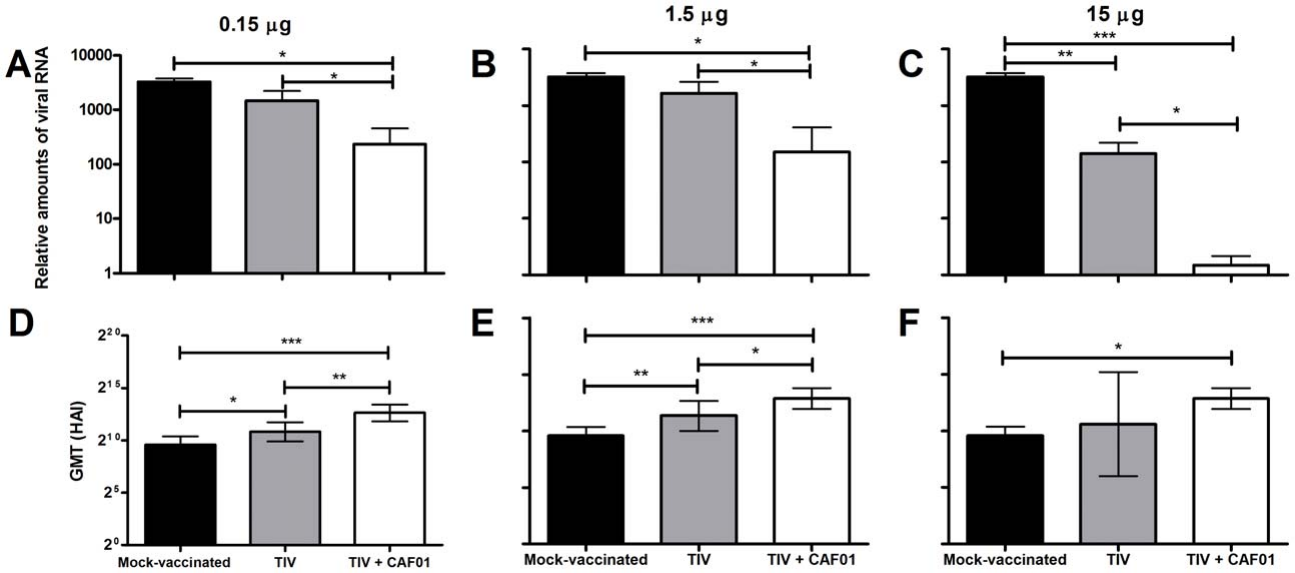
Challenge with A/Brisbane/59/2007 (H1N1) after immunizations with different doses. 6 to 10 months-old ferrets were immunized twice at two week-intervals with CAF01 adjuvanted and non-adjuvanted influenza vaccine at different dose levels. A third group was left un-vaccinated. All ferrets were challenged with 10^7^ TCID_50_ of A/Brisbane/59/2007 (H1N1) four weeks after the second immunization (n = 4). **A**, **B** and **C** show the relative amounts of viral RNA found in nasal washes of individual infected animals at peak replication day (day 4), measured by quantitative RT-PCR. **D**, **E** and **F** show hemagglutination inhibition assay serum titers at day 11 using influenza A/Brisbane/59/2007. Values marked with an asterisk are significantly different (*, *p*<0.05; **, *p*<0.01; ***, *p*<0.001), assessed by ANOVA.

Four weeks after the second immunization, the animals received a H1N1 challenge through the nasal cavities. Nasal washes were performed at day 4 post-challenge and the relative amounts of viral RNA in each wash were measured by quantitative RT-PCR (fig. 4A, B and C). At all three dose levels, the CAF01-adjuvanted vaccine was the best-performing vaccine, giving rise to significantly ower levels of virus excretion even at a low dose of 0.15 µg of vaccine. At this dose level (0.15 µg adjuvanted with CAF01), the reduction in viral RNA load was comparable to that observed with the non-adjuvanted 15 µg dose. The highest level of protection was obtained with 15 µg of vaccine in CAF01 which led to a 100-fold reduction of the viral load compared to the same dose of vaccine without CAF01 (fig. 4C). Finally, HAI titers were measured in serum samples during infection and further supported that CAF01 enhanced the effect of the TIV with all three dose levels giving rise to significantly higher HAI titers compared to the mock-vaccinated animals (fig. 4D, E and F).

### CAF01 compares favourably to a squalene emulsion based adjuvant

**Figure 5:**
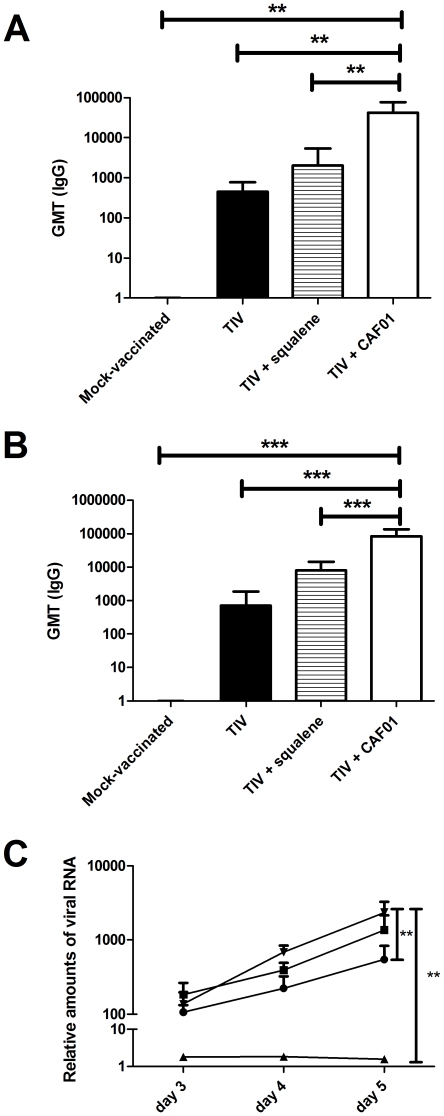
Vaccine-specific IgG antibodies after immunizations with different TIV formulations. 6 to 10 months-old ferrets were immunized twice at four week-intervals with different doses of CAF01 adjuvanted, squalene emulsion adjuvanted, and non-adjuvanted influenza vaccine Vaxigrip season 2008/2009. **A.** vaccine-specific IgG titers measured in serum by ELISA two weeks after the first immunization. **B.** vaccine-specific IgG titers measured in serum by ELISA two weeks after the second immunization. All ELISA values are expressed as geometric mean titers (GMT) and values marked with an asterisk are significantly different (*, *p*<0.05; **, *p*<0.01; ***, *p*<0.001), assessed by ANOVA. **C.** Relative amounts of viral RNA in nasal washes day 4 after challenge of ferrets immunized with different TIV formulations: TIV + CAF01 (▴), squalene + TIV (•), TIV alone (▪) or mock-vaccinated (▾). All ferrets were challenged with 10^7^ TCID_50_ of A/Brisbane/59/2007 (H1N1). (*, *p*<0.05; **, *p*<0.01; ***, *p*<0.001).

Since squalene emulsions are being increasingly used as adjuvants for influenza vaccines, we have compared the effects on CAF01 to that of a custom-made squalene emulsion with a composition based on available information from the adjuvanted prepandrix vaccine [27]. Ferrets received two injections of TIV (15 µg of HA per dose) either without adjuvant, adjuvanted with CAF01, or adjuvanted with the squalene emulsion. A mock-vaccinated control group received only PBS injections. Four weeks after the first injection, animals which received the vaccine adjuvanted with the squalene emulsion had higher vaccine-specific serum IgG titers than the animals which received the vaccine alone (fig. 5). At the same timepoint, the animals that received the vaccine adjuvanted with CAF01 had antibody titers higher than the squalene emulsion group (p<0.01). These trends were confirmed after the second injection with animals that received CAF01 displaying significantly higher titers of serum IgG than those which received squalene emulsion-adjuvanted TIV or TIV without adjuvant.

Four weeks after the second injection, the animals were challenged intra-nasally with a homologuous influenza virus. Nasal samples taken during the peak replication of the virus (day 4–5) showed that the squalene emulsion reduced the excretion of viral RNA by 60 to 75% (p<0.01) compared to animals that did not receive any vaccine, and by more than 50% compared to the animals that received the TIV without adjuvant (fig. 5C). Importantly, animals that received the TIV adjuvanted with CAF01 had very low levels (close to the detection level) of viral RNA in their nasal washes compared to control animals.

## Discussion

This study demonstrates that the novel liposomal adjuvant CAF01 potentiates the humoral response and adds an additional CMI response to the response promoted by a commercially available split vaccine. This adjuvantation of the vaccine results in accelerated clearance and influenza virus titers in the nasal cavities of ferrets were reduced by two to three orders of magnitude compared to non-vaccinated animals.

Influenza A virus infections in mammals are characterized by a very prompt immune response that can clear the virus 5 to 7 days after infection [35]. Unlike many other infections, antibody response is detectable already within the first week after infection, noticeably with the presence of IgM in the blood and IgA in the nasal mucus [36]. In spite of the limitations of a response based on only humoral immunity, antibodies are still the main response induced by influenza vaccines [9]. The CAF01 adjuvant potentiates the production of antibodies even after a single immunization and both in terms of serum IgG and mucosal IgA antibodies. CAF01 induces a quantitatively larger humoral immune response than the conventional vaccine alone and also promote a much stronger potentiation of the response than a squalene emulsion adjuvanted vaccine. In addition to increasing the antibody response, CAF01 also affects the CMI response whereas no such response was seen in ferrets receiving non-adjuvanted split vaccine. A major improvement to the current influenza vaccines would be an vaccine with the ability to promote a T-cell response that could potentially lead to cross-protection between heterologous strains [37]. Although such a cross-protection most likely would operate more efficiently through T-cell responses directed against conserved proteins located in the inner part of the virion rather than the highly variable surface proteins hemagglutinin and neuraminidase present in the vaccine used herein, the induction of IFN-γ positive lymphocytes upon specific stimulation with recombinant H1 demonstrates that CAF01 has the ability to generate potent CMI responses (fig. 1C). As there is accumulating evidence that CMI responses correlates to protection against influenza [38], there is a need to expand and optimize ferret specific reagents. Some recent progress have been made in this area, including the development of an IFN-γ ELISPOT assay [39] as well as flow cytometry assays [39], [40]. These will allow for a deeper investigation of the importance of the CMI response in providing cross-protection and long-lived immunity.

In the present study we used the overall level of infection driven lymphocyte activation by stimulating PBMCs with a polyclonal activator (PMA and ionomycin) known to expand preferentially activated T cells in a TCR-independent manner [33], [34]. Virus-specific lymphocytes make up for the largest part of the new lymphocytes appearing during acute infections [32]. Hence it is here assumed that the increase in IFN-γ producing lymphocytes after challenge reflects increased numbers of influenza-specific T-cells as a consequence of progressive viral infection. Given the importance of NK cells in controlling the virus during the early days of the infection [41], [42], it is also possible that a significant part of these IFN- γ producing lymphocytes might be NK cells. The non-vaccinated group and the group that received the vaccine were both characterized by a higher percentage of IFN-γ producing cells during the later stages of infection than the group receiving the vaccine adjuvanted with CAF01 (fig. 2E). The absence of this population of IFN-γ producing cells during infection in ferrets vaccinated with CAF01/influenza vaccine suggests that the immunity conferred by influenza-specific antibodies maintained the infection at low levels.

Most influenza vaccination studies in ferrets use parameters such as weight loss and temperature to evaluate the protection conferred by vaccines. Clinical scores rely on the similarity in symptoms, severity and course of the disease between humans and the ferret model to assess the clinical potential of vaccines. However, it has been observed in ferrets that different H1N1 strains of influenza induce fever and other constitutional effects with different severity [43]. As A/New Caledonia/20/99 (H1N1) and A/Brisbane/59/2007 (H1N1) produce only a mild disease in ferrets and humans, it was not possible to use differences in weight loss as an indicator of protection. Similarly, rectal temperature measurements were inconclusive and the method has been criticized in several reports [44], [45], and subcutaneous chips for continuous monitoring of the temperature is probably necessary if this parameter is to be employed in the ferret model [46]. In the present study it was chosen to focus on viral excretion and the ability to inhibit viral growth as the most reliable indicators of protection.

A major finding of these experiments was the dose-sparing effect of CAF01 on the commercial split-vaccine (fig. 4). Animals that received 0.15 µg TIV vaccine adjuvanted with CAF01 had higher antibody titers and controlled viral replication as efficiently as animals vaccinated with 15 µg of the non-adjuvanted vaccine. It is interesting to note in this experiment that there is no strict correlation between the improvement of the antibody response induced by the vaccine and the reduction of the viral load in challenged animals. Animals that received CAF01 with 0.15 µg of the vaccine had IgG, IgA and HAI titers at the same overall level as animals that received 15 µg of the vaccine with CAF01, yet the latter displayed lower viral load in their nasal cavities (fig. 4).

Finally, in a head-to-head comparison with a squalene emulsion with a composition similar to the adjuvant in the Prepandrix vaccine, CAF01 adjuvanted TIV proved to be both more immunogenic (fig. 5) and better at reducing the excretion of viral RNA in infected animals (fig. 5C).

The mechanism by which CAF01 improves the efficacy of the commercial inactivated influenza vaccines still needs to be analysed in more detail. Evidence from *in vivo* and *in vitro* systems suggests that the mechanism relies on the fine-tuned combination of an efficient delivery system (DDA) [47] and the potent immunomodulator (TDB) [24]. DDA also forms a depot at the injection site which is important for obtaining long-lived memory responses [48]. In a recent mouse study, CAF01 was found to induce robust CMI responses maintained for more than one year [49]. TDB is a synthetic immunomodulator signalling through the Syk-Card9 pathway and inducing robust combined Th1 and Th17 responses [50]. As a recent study suggest that subsets of IL-17 producing T-cells might have an important role in the immune response to influenza virus [51], this distinct characteristic of CAF01 could be highly relevant in an influenza vaccine. Due to a lack of reagents, it is not possible to investigate the role of Th17 responses in the ferret model.

The observation that CAF01 induces a strong dose-sparing effect, including long-lived and combined humoral and cellular responses, warrants further investigation of the potential of CAF01 as adjuvant in influenza vaccines.
